# Inflammation, mechanical allodynia and an exaggerated exercise pressor reflex during disease progression in UCD‐type 2 diabetes mellitus rats

**DOI:** 10.1113/EP093516

**Published:** 2026-05-05

**Authors:** Michelle L. Harrison, Richard K. McCuller, Yu Huo, Amshula N. Gajula, Kai Ybarbo, Mark J. Van Ryzin, Kimber L. Stanhope, Peter J. Havel, Audrey J. Stone

**Affiliations:** ^1^ Department of Kinesiology and Health Education The University of Texas at Austin Austin Texas USA; ^2^ Department of Cardiovascular Medicine Mayo Clinic Rochester Minnesota USA; ^3^ Department of Molecular Biosciences, School of Veterinary Medicine and Department of Nutrition University of California Davis California USA; ^4^ Department of Cellular and Physiological Sciences School of Medicine University of British Columbia Vancouver British Columbia Canada

**Keywords:** blood pressure, C‐reactive protein, cytokine

## Abstract

Chronic low‐grade inflammation characterizes type 2 diabetes mellitus (T2DM) and underlies the development of peripheral neuropathy, symptoms of which include mechanical allodynia and an exaggerated exercise pressor reflex. The purpose of this study was to determine whether mechanical allodynia and/or specific concentrations of inflammatory mediators coincide with the presence of an exaggerated exercise pressor reflex. Haemoglobin A1c was assessed to detect diabetes onset (threshold ≥5.6%) in male University of California Davis (UCD)‐T2DM rats (*n* = 18). Age‐matched, healthy Sprague–Dawley rats (*n* = 18) served as controls. Monthly measurements assessed mechanical allodynia (via paw withdrawal threshold), insulin and inflammatory mediators (via ELISA and multiplex kits). Paw withdrawal threshold was not different between groups prior to diabetes onset. However, by 8 weeks post‐onset, T2DM rats demonstrated a significant decrease in pain threshold compared to that before diabetes onset, which persisted for 8 weeks. This is the same period in diabetes progression as when the exercise pressor reflex is exaggerated. Prior to diabetes onset, UCD‑T2DM rats had significantly higher circulating levels of IL‑6 and IL‑1β, and these elevations persisted throughout disease progression, with only CRP showing a significant increase at 8 weeks post‑onset. These findings offer a useful foundation for further exploration into the assessments of mechanical allodynia and inflammation to determine when those with T2DM are at an increased risk for experiencing adverse cardiovascular events.

## Abstract (highlights)

- **What is the central question of this study?**
  Does mechanical allodynia and/or a specific inflammatory profile coincide with the presence of an exaggerated exercise pressor reflex in type 2 diabetes mellitus (T2DM) rats?
- **What is the main finding and its importance?**
  Elevated systemic inflammation and mechanical allodynia are both present when the exercise pressor reflex is exaggerated during T2DM, suggesting that further investigation of these two mechanisms may lead to new approaches for evaluating an old disease.

## 1 INTRODUCTION

Type 2 diabetes mellitus (T2DM) is characterized by chronic low‐grade inflammation and is a major risk factor for peripheral neuropathy and cardiovascular disease (Dhurandhar et al., 2025; Donath & Shoelson, 2011; Galicia‐Garcia et al., 2020). Elevated levels of pro‐inflammatory cytokines, including interleukin (IL)‐6, IL‐1β, and tumour necrosis factor‐α (TNF‐α), have been linked to both peripheral neuropathy and altered autonomic regulation of circulation during exercise, particularly through the exercise pressor reflex (Copp et al., 2015; Huo et al., 2024; Leung & Cahill, 2010; Xing et al., 2018). T2DM progresses over time, producing dynamic changes in blood pressure regulation and sensation mediated by thinly myelinated Aδ fibres (group III afferents) and unmyelinated C‐fibres (group IV afferents) (Huo et al., 2022; Sveen et al., 2013). However, whether the cutaneous afferents associated with peripheral neuropathy are affected at the same period of disease progression as the muscle afferents involved in the exercise pressor reflex has yet to be explored.

Diabetic peripheral neuropathy (DPN) causes mechanical allodynia in those with T2DM by affecting thinly myelinated afferents (Khan et al., 2002), where sensitization or damage to peripheral nerve endings produces pain in response to previously non‐painful stimuli. This heightened sensory response parallels the exaggerated blood pressure increase evoked by group III and IV afferent activity during skeletal muscle contraction, namely the exercise pressor reflex (McCloskey & Mitchell, 1972b). Although DPN symptoms are well recognized and routinely assessed clinically, the exaggerated exercise pressor reflex and its cardiovascular risks are less understood. During muscle contraction, this reflex increases sympathetic and decreases parasympathetic activity, raising heart rate, myocardial contractility and blood pressure (Alam & Smirk, 1937; Coote et al., 1971; McCloskey & Mitchell, 1972a). This reflexive response is necessary to redirect blood flow to working skeletal muscle. However, when the reflex is exaggerated, blood pressure rises to dangerous levels, increasing the risk of myocardial infarction or stroke (Kurl et al., 2001; Laukkanen et al., 2006). Exaggerated blood pressure responses to exercise have been observed in individuals with T2DM (Holwerda et al., 2016; Regensteiner et al., 2009; Scott et al., 2008) and in T2DM rat models (Estrada et al., 2024; Grotle et al., 2019; Huo et al., 2022, 2024; Kim et al., 2019). Mechanical allodynia and an exaggerated exercise pressor reflex likely develop concurrently during T2DM progression, implying widespread involvement of thinly myelinated afferents.

Both pro‐ and anti‐inflammatory mediators play key roles in the pathophysiology of T2DM, cardiovascular disease and DPN. Elevated levels of TNF‐α, IL‐1β and IL‐6 are observed in individuals at risk for T2DM, and inhibiting these cytokines improves glycaemic control (Spranger et al., 2003) and reduces pain (Klimek et al., 2016). In a rat model of localized inflammation, cytokine response varies (Xie et al., 2006), suggesting that T2DM progression may involve dynamic shifts in inflammatory mediator profiles, which could have diagnostic significance. A previous study found that injecting IL‐6 into the hindlimb of healthy rats exaggerates the exercise pressor reflex (Copp et al., 2015). Furthermore, elevated concentrations of IL‐6, TNF‐α and IL‐1β are associated with progressive nerve degeneration (Doupis et al., 2009). What remains unknown is whether there is an inflammatory profile that emerges concurrently with mechanical allodynia and an exaggerated exercise pressor reflex.

As diabetes progresses, symptoms of DPN emerge, including mechanical allodynia and an exaggerated exercise pressor reflex, both of which are linked to chronic low‐grade inflammation (Cheng et al., 2024; Copp et al., 2015). The purpose of this study was to determine whether mechanical allodynia and/or a specific inflammatory profile coincides with the presence of an exaggerated exercise pressor reflex. A previous study reported this exaggerated reflex in T2DM rats 7–15 weeks after disease onset (Huo et al., 2022). We hypothesized that, during this same period, mechanical threshold would be lower, pro‐inflammatory cytokines would be elevated and anti‐inflammatory cytokines would be decreased.

## 2 METHODS

All procedures were approved by the Institutional Animal Care and Use Committee of The University of Texas at Austin (Protocol AUP‐2022‐0049), which adheres to the USDA Animal Welfare act and NIH Office of Laboratory Animal Welfare policies.

Adult male University of California Davis‐type 2 diabetes mellitus (UCD‐T2DM) rats were used in the study (*n* = 18). This model was chosen because rats develop T2DM over time, paralleling the pathophysiology of the disease in humans (Cummings et al., 2008; Kleinert et al., 2018). The incidence of T2DM in this model is much lower in females and the onset is at a much older age; thus, only male rats were included. Healthy, age‐matched male Sprague–Dawley (Charles River, Wilmington, MA, USA) rats served as controls (*n* = 18). Rats were housed in a temperature‐controlled room (24 ± 1°C) with a 12:12 h light‐dark cycle and fed a standard rodent diet (Purina Mills Lab Diet #5053, Purina Animal Nutrition LLC, Arden Hills, MN, USA) and tap water ad libitum.

To accurately capture diabetes onset, starting at 10 weeks of age, biweekly measures of haemoglobin A1c (HbA1c; A1CNow+, PTS diagnostics, Indianapolis, IN, USA) were taken and a HbA1c ≥ 5.6% (Samora et al., 2023) was diagnostic.

Every 4 weeks (4 weeks pre to 24 weeks post diabetes onset), rats were weighed, tail vein blood was drawn, and paw withdrawal threshold (PWT), an indicator of mechanical allodynia, was assessed. Inflammatory mediators were quantified in serum using multiplex kits [RADPKMAG (neat) and RECYTMAG (diluted ×4), Millipore, Burlington, MA, USA] and an ELISA kit for C‐reactive protein (CRP) [Invitrogen ECCRP (diluted ×50,000), Thermo Fisher Scientific, Waltham MA, USA]. Standards, controls and samples were measured in duplicate according to the manufacturer's instructions.

Rats were acclimated to the von Frey chamber and von Frey hair (VFH) monofilaments (North Coast Medical, Morgan Hill, CA, USA) were used to assess PWT using a validated up–down method (Detloff et al., 2010). An online algorithm was used to convert each VFH (in grams) to 50% PWT (Christensen et al., 2020). Since pain sensation is perceived on a logarithmic scale, PWT data were log transformed for parametric analysis (Mills et al., 2012). Following data collection, rats were euthanized with an overdose of isoflurane followed by an intravenous injection of saturated potassium chloride (>200 mg/kg). Due to the complexity of assessing the exercise pressor reflex, repeat measures are not possible. The current study involves only those measurements that were repeated over time.

### 2.1 Statistics

Data are presented as means (SD). A two‐tailed, unpaired Student's *t*‐test was used to compare baseline concentrations between groups. Data collected every 4 weeks were analysed using a mixed‐method two‐way ANOVA with time repeated in which disease and time were main factors. When indicated, Holm–Sidak *post hoc* analysis was used to compare each time point to 4 weeks prior to disease onset within each group and to compare between groups for each time point. Significance was set a priori at *P *< 0.05. All statistical analyses and figures were created using Prism (v.10.3.1, GraphPad Software, Boston, MA, USA).

## 3 RESULTS

**FIGURE 1:**
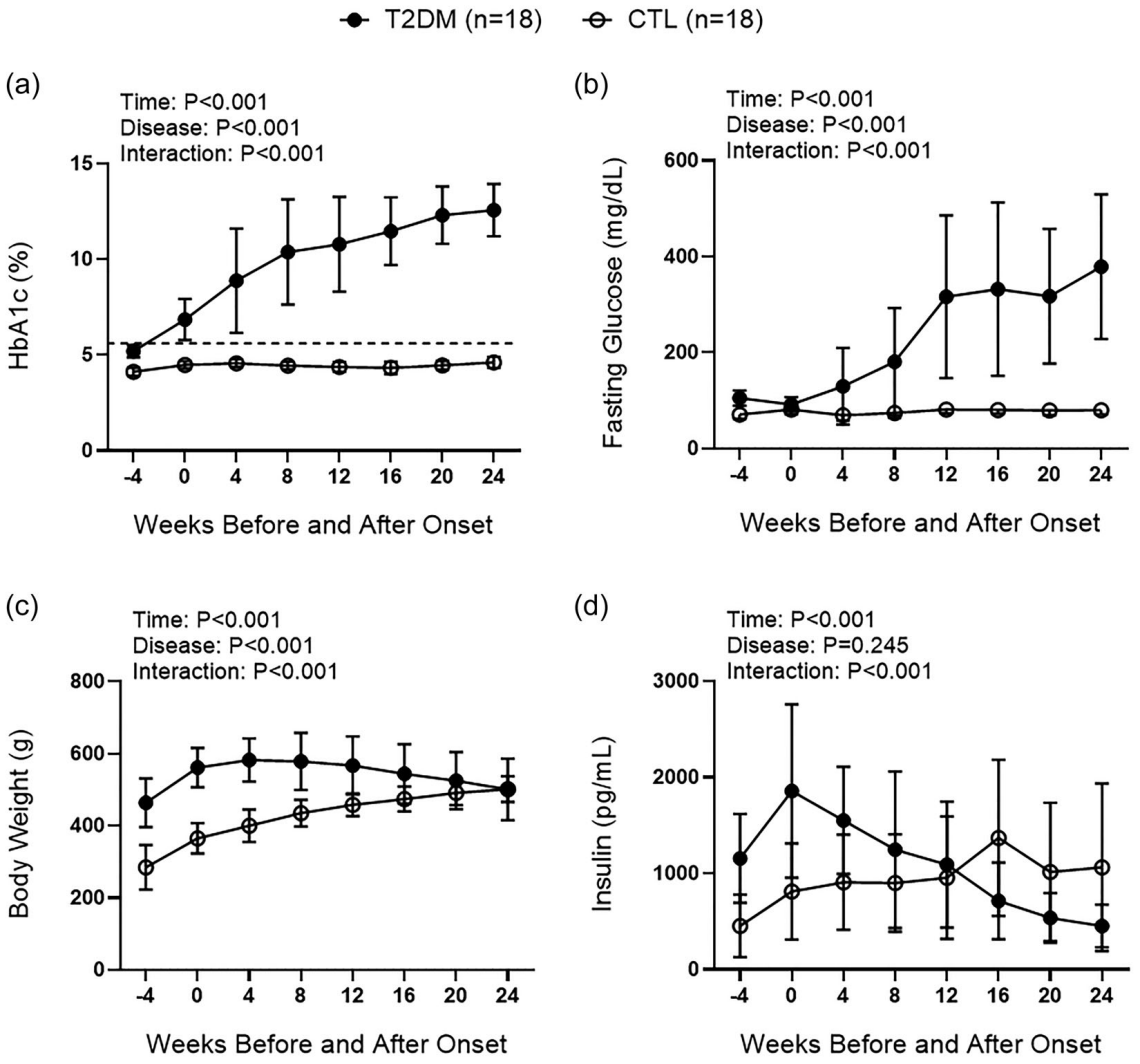
Progression of diabetes in untreated male UCD‐T2DM (*n* = 18) and control rats (*n* = 18). Data are presented as means (SD) and were analysed using a two‐way repeated measures ANOVA. Diabetes onset is marked at week 0; −4 indicates 4 weeks prior, and data are shown through 24 weeks post‐onset. Dashed line indicates 5.6%, the criterion for diagnosis. CTL, age‐matched healthy control; T2DM, Type 2 diabetes mellitus.

As expected, both HbA1c and glucose increased over time. Body weight decreased as T2DM progressed untreated, and insulin initially increased as tissues became resistant and then decreased as pancreatic decompensation occurred (Figure 1a–d and Supporting information, Tables S1 and S4).

**FIGURE 2:**
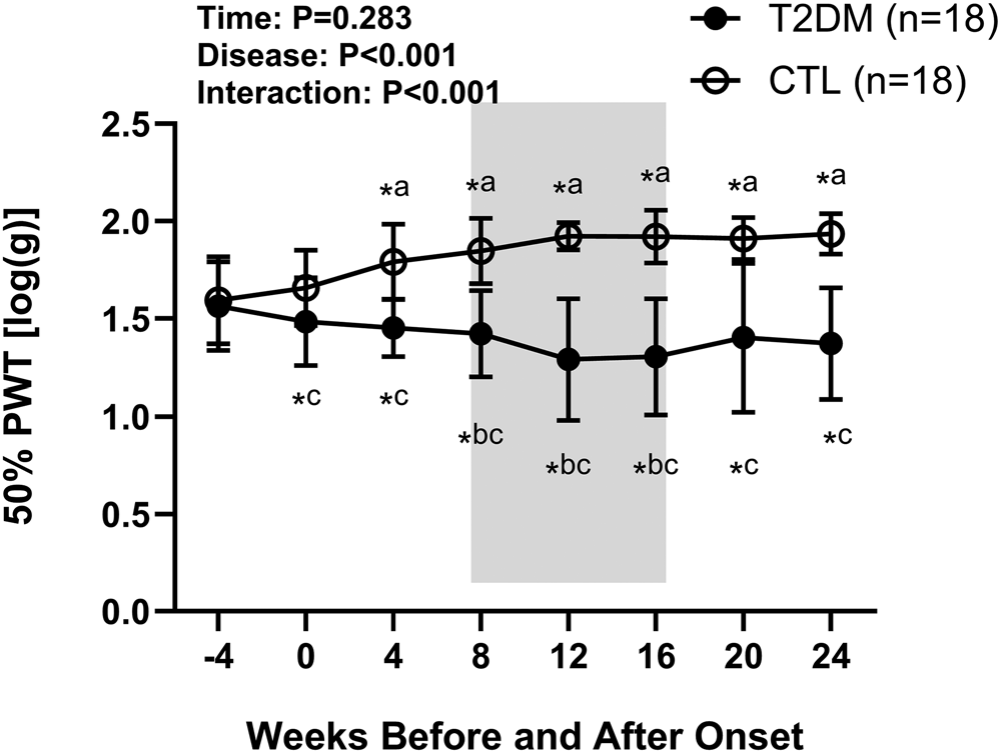
Onset and progression of mechanical allodynia in male UCD‐T2DM (*n* = 18) and control rats (*n* = 18). Data are presented as means (SD) and were analysed using a mixed‐method two‐way ANOVA with time repeated and Holm–Sidak for *post hoc* analyses. Diabetes onset is marked at week 0; −4 indicates 4 weeks prior, and data are shown through 24 weeks post‐onset. The shaded region represents the period when the exercise pressor reflex is exaggerated (7–15 weeks post‐onset, previously published findings). *P < 0.05, (a) compared to −4 weeks within CTL, (b) compared to −4 weeks within T2DM, (c) compared to control at same time point (see Supporting information, Table S5 for all *P*‐values). CTL, age‐matched healthy control; PWT, paw withdrawal threshold; T2DM, type 2 diabetes mellitus.

Paw withdrawal threshold did not differ between groups prior to diabetes onset (Figure 2 and Supporting information, Tables S2 and S5). Compared with 4 weeks before onset, T2DM rats demonstrated a trending decrease in pain threshold that became significant only from 8 to 16 weeks post onset [−4 weeks: 1.56 (0.23); 8 weeks: 1.42 (0.22), *P* = 0.009; 12 weeks: 1.29 (0.31), *P* = 0.003; and 16 weeks: 1.30 (0.30), *P* = 0.009; log(g)]. This is the same period in disease progression as when the exercise pressor reflex is exaggerated (Huo et al., 2022). Compared with 4 weeks before onset, pain threshold increased over time in healthy rats (−4 weeks: 1.59 (0.22) vs 4 weeks: 1.79 (0.20), 8 weeks: 1.85 (0.17), 12 weeks: 1.92 (0.07), 16 weeks: 1.92 (0.14), 20 weeks: 1.91 (0.11), and 24 weeks: 1.94 (0.11); log(g); *P *< 0.001 for all). When comparing PWT between groups, within each time point, T2DM rats had significantly lower thresholds than CTL rats starting at disease onset (0 week: *P* = 0.018 and 4 weeks, 8 weeks, 12 weeks, 16 weeks, 20 weeks and 24 weeks: *P *< 0.001 for all, Supporting information, Tables S2 and S5).

**FIGURE 3:**
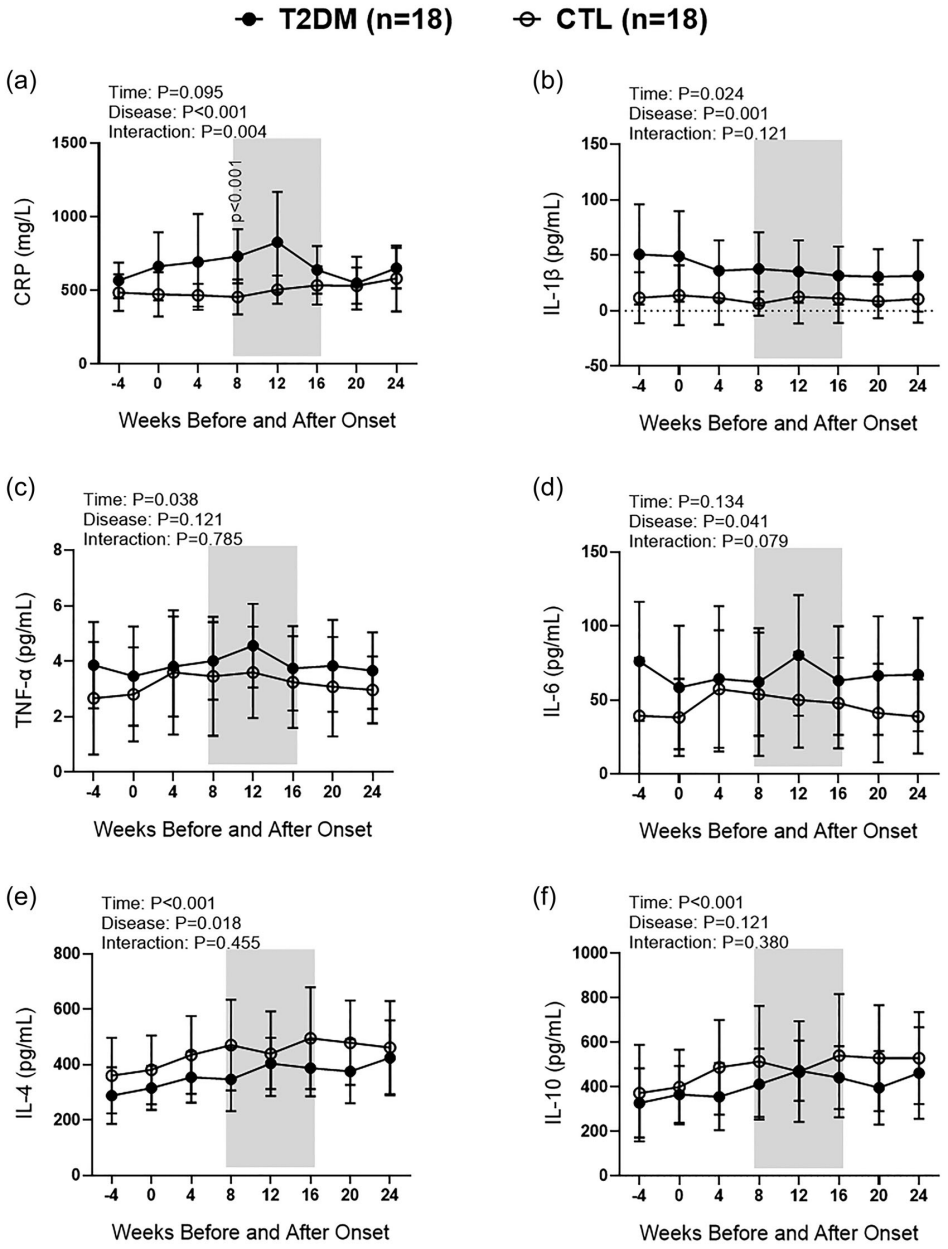
Changes in circulating inflammatory mediators with T2DM progression in male UCD‐T2DM (*n* = 18) and control rats (*n* = 18). Data are presented as means (SD) and were analysed using a two‐way repeated measures ANOVA and Holm–Sidak for *post hoc* analyses. Diabetes onset is marked at week 0; −4 indicates 4 weeks prior, and data are shown through 24 weeks post‐onset. The shaded region represents the period when the exercise pressor reflex is exaggerated (7–15 weeks post‐onset, previously published). CRP, C‐reactive protein; CTL, age‐matched healthy control; IL, interleukin; T2DM, type 2 diabetes mellitus; TNF, tumour necrosis factor.

Baseline characteristics were compared between groups and are presented in Table 1. Though not yet diabetic, the T2DM group had a higher HbA1c, were significantly heavier and were already insulin resistant. In addition, IL‐6 and IL‐1β concentrations were significantly greater in T2DM rats. IL‐6 and IL‐1β were higher, and IL‐4 remained lower in the T2DM rats compared with controls, but no cytokine concentration changed significantly as diabetes progressed (Figure 3b–f and Supporting information, Tables S3 and S6). C‐reactive protein, a general indicator of systemic inflammation, trended upwards and became significantly increased at 8 weeks post‐onset (*P* = 0.027) (Figure 3a and Supporting information, Tables S3 and S6), the same period as when the exercise pressor reflex is exaggerated.

**TABLE 1:** Comparisons between male T2DM and CTL before diabetes onset.

	T2DM (*n* = 18)	CTL (*n* = 18)	*P*
HbA1C (%)	5.2(0.3)	4.1(0.2)	<0.001
Body weight (g)	463(68)	284(62)	<0.001
Insulin (pg/mL)	1157(462)	454(325)	<0.001
Paw withdrawal threshold (log(g))	1.56(0.24)	1.60(0.22)	0.564
IL‐4 (pg/mL)	288(102)	360(137)	0.083
IL‐10 (pg/mL)	328(156)	372(217)	0.487
IL‐6 (pg/mL)	76(40)	39(39)	0.009
IL‐1β (pg/mL)	51(45)	12(23)	0.002
TNF‐α (pg/mL)	3.9(1.60)	2.7(2.0)	0.057
CRP (mg/L)	565(122)	483(125)	0.055

Abbreviations: CRP, C‐reactive protein; CTL, age‐matched healthy control; IL, interleukin; T2DM, type 2 diabetes mellitus; TNF, tumour necrosis factor.

## 4 DISCUSSION

This study was the first to longitudinally examine circulating inflammatory markers alongside measures of mechanical allodynia from before disease onset through the progression of T2DM, highlighting novel avenues for future cardiovascular risk evaluation. Our primary finding was that mechanical allodynia is present at the same time (by 8 weeks post‐onset) as when the exercise pressor reflex is exaggerated in T2DM rats, suggesting that individual perception of mechanical allodynia may be an indicator of abnormal reflexive cardiovascular responses to exercise. Inflammation was also greater at this time, indicated by elevated CRP concentrations in T2DM compared to before onset. Contrary to our hypothesis, no differences were found in any cytokines specifically during this period. Interestingly, both IL‐1β and IL‐6 concentrations were already higher in T2DM rats before the onset of the disease and remained elevated over time. Furthermore, IL‐4 remained lower in T2DM compared to healthy rats.

Mechanically sensitive afferents that contribute to mechanical allodynia and the exercise pressor reflex are altered in T2DM. Several studies determined that neuropathy varies from hypersensitivity to numbness depending on duration and severity of disease (Çakici et al., 2016). This study demonstrated a similarly fluctuating pattern in the mechanical threshold of T2DM rats, where it decreased (indicating hypersensitivity) for only 8–16 weeks after the onset of the disease compared to baseline normo‐sensitivity. This suggests that the observed mechanical allodynia is present at the same period in the disease (i.e., 8–16 weeks post onset) as when the exercise pressor reflex is exaggerated (Huo et al., 2022, 2024). Importantly, these findings pertain to baseline sensitivity for each individual rat, allowing for individual perception of intensity, commonly assed using the visual analog scale in patients with mechanical allodynia (Peyron et al., 2004). One limitation of this study is that the exercise pressor reflex could not be tested in the same rats that assessed longitudinal measurements of inflammatory markers and mechanical allodynia. Since differences in mechanical pain threshold were detected between groups beginning at disease onset, our findings also suggest that mechanical allodynia occurs before any documented exaggeration in the exercise pressor reflex in T2DM. Further studies are needed to determine the earliest time point at which the exercise pressor reflex is exaggerated in T2DM in relation to when mechanical allodynia emerges in healthy controls.

Interestingly, CRP was the only inflammatory mediator that was significantly different from baseline concentrations during the same period (i.e., 8–16 weeks post‐onset) as exaggerated exercise pressor responses (Huo et al., 2022). This increase in CRP was transient, suggesting that further inquiry is needed to determine possible downstream effects of a surge in CRP. Pro‐inflammatory cytokines IL‐1β and IL‐6 were higher in T2DM before disease onset and throughout the progression of the disease, regardless of the time point. These findings are similar to those in Huo et al., where IL‐1β concentration was significantly greater in T2DM rats with an exaggerated exercise pressor reflex compared to controls (Huo et al., 2024). Previous studies in humans have demonstrated that increases in visceral fat increase pro‐ and decrease anti‐inflammatory cytokines (Hernandez et al., 2024; Kolb, 2022; Rakotoarivelo et al., 2018; Wueest & Konrad, 2020). Notably, before onset T2DM rats in the current study had similar body weights to the pre‐diabetic rats in Samora et al. (2023) and, although visceral fat was not measured in this study, it is likely that T2DM rats had greater visceral fat than the healthy controls. This might explain the higher concentrations of IL‐1β and IL‐6 before disease onset.

Chronic low‐grade inflammation is characteristic of individuals with T2DM and contributes to mechanical allodynia and exaggerated pressor responses to exercise. The present study is the first to demonstrate that mechanical allodynia is present at the same time that the exercise pressor reflex is exaggerated in T2DM; however, it is still not known if mechanical allodynia can be used to determine risk of cardiovascular events in this clinical population. Moreover, this study found that systemic inflammation was also significantly elevated during the same point in the progression of T2DM when the exercise pressor reflex is exaggerated. Notably, this was reflected by increased CRP concentrations rather than a specific inflammatory profile involving pro‐ and/or anti‐inflammatory cytokines. These findings offer a useful foundation for further exploration into the assessments of mechanical allodynia and inflammation to determine when those with T2DM are at an increased risk for experiencing adverse cardiovascular events.

## AUTHOR CONTRIBUTIONS

Michelle L. Harrison and Audrey J. Stone: conception, design, interpretation of data, drafting of manuscript; Michelle L. Harrison, Richard K. McCuller and Yu Huo: data collection; Michelle L. Harrison, Amshula N. Gajula, Kai Ybarbo and Mark J. Van Ryzin: data analysis; Amshula N. Gajula and Michelle L. Harrison: figures; all authors edited manuscript. All authors have read and approved the final version of this manuscript and agree to be accountable for all aspects of the work in ensuring that questions related to the accuracy or integrity of any part of the work are appropriately investigated and resolved. All persons designated as authors qualify for authorship, and all those who qualify for authorship are listed.

## CONFLICT OF INTEREST

The authors declare no conflicts of interest.

## Supporting information



Tables S1–S6.

## Data Availability

The data are available from the corresponding author upon reasonable request.
